# Diagnostic stability of 346 patients with borderline personality disorder based on retrospective clinical records

**DOI:** 10.1192/j.eurpsy.2023.1302

**Published:** 2023-07-19

**Authors:** J. Henriques-Calado, M. M. Schumacher, J. Gama Marques

**Affiliations:** 1 Faculdade de Psicologia, Universidade de Lisboa, Alameda da Universidade, 1649-013 Lisboa, Portugal; 2 CICPSI, Faculdade de Psicologia, Universidade de Lisboa, Alameda da Universidade, 1649-013 Lisboa, Portugal; 3 Consultant, 4450 Sissach, Switzerland; 4 Consulta de Esquizofrenia Resistente, Hospital Júlio de Matos, Centro Hospitalar Psiquiátrico de Lisboa, Avenida do Brasil, 53, 1749-002 Lisboa, Portugal; 5 Clínica Universitária de Psiquiatra e Psicologia Médica, Faculdade de Medicina, Universidade de Lisboa, Avenida Professor Egas Moniz, 1649-028 Lisboa, Portugal

## Abstract

**Introduction:**

State-of-the-art research highlights that borderline personality disorder have high rates of comorbid Axis I disorders, which imply uncertainty in establishing an accurate diagnosis and can be some of the most challenging patients for clinicians and researchers.

**Objectives:**

This study seeks to observe the diagnostic stability in borderline personality disorder patients, in order to increase empirical knowledge through a retrospective look at the historical line of diagnoses.

**Methods:**

A twenty-year retrospective study at a psychiatric hospital, searching at the electronic clinical records for all patients with borderline personality disorder diagnosis, under the code 301.83 from World Health Organization’s International Classification of Diseases, 9^th^ Revision (WHO ICD9). A 346 patients’ sample was identified aged between 18 and 83 years (*M*_age_=44.14 years, *SD*=11.18; predominantly female 73.70%; *M*_schooling_=9.31years; *M*_admissions_=4.72_times_, *SD*=9.21; 2^nd^-5^th^ comorbid diagnosis, a 75.72% sample with three diagnosis); excluding organic cerebral syndrome and no comorbidity besides drug abuse, or no comorbidity at all.

**Results:**

As a general observation, the following diagnoses are indicated: 44.09% major depressive disorder, 33.16% affective disorder, 13.05% schizophrenia, and 9.70% mania. As a spectrums disorders analysis (Figure 1), differential percentage occurrences are identified in patients with borderline personality disorder.

**Image:**

**Figure:**
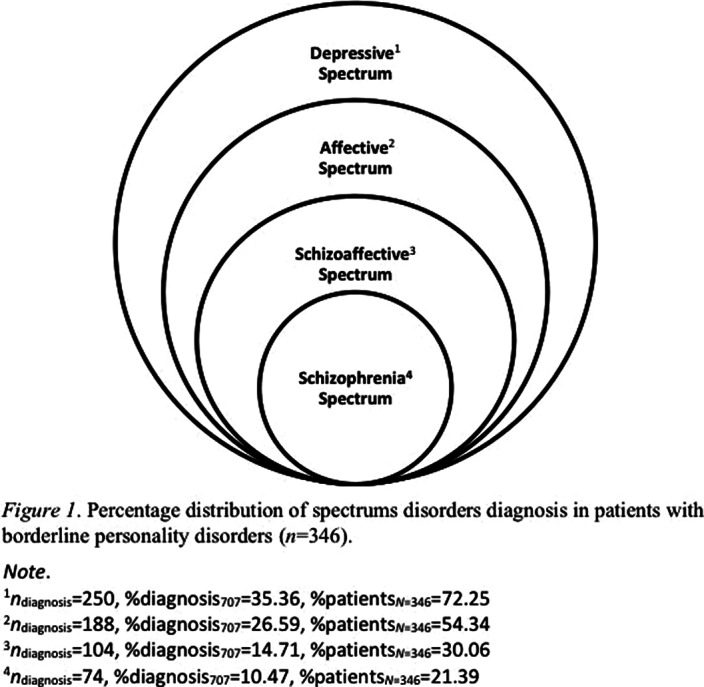

**Conclusions:**

Based on clinical diagnoses records of borderline personality disorder patients, some spectrums disorders are highlighted, to be reported in descending order of incidence: depressive, affective, schizoaffective and schizophrenia spectrums.

**Disclosure of Interest:**

None Declared

